# Probing Protein Folding with Sequence-Reversed α-Helical Bundles

**DOI:** 10.3390/ijms22041955

**Published:** 2021-02-16

**Authors:** Aikaterini Kefala, Maria Amprazi, Efstratios Mylonas, Dina Kotsifaki, Mary Providaki, Charalambos Pozidis, Melina Fotiadou, Michael Kokkinidis

**Affiliations:** 1 Institute of Molecular Biology and Biotechnology, Foundation for Research and Technology–Hellas (IMBB-FORTH), 70013 Heraklion, Greece; kefalaka@uoc.gr (A.K.); amprazi@imbb.forth.gr (M.A.); stratos_mylonas@imbb.forth.gr (E.M.); dkotsifa@imbb.forth.gr (D.K.); providak@imbb.forth.gr (M.P.); pozidis@imbb.forth.gr (C.P.); 2 Department of Biology, University of Crete, 70013 Heraklion, Greece; melina_fotiadou@yahoo.gr

**Keywords:** protein folding, α-helix, coiled-coil proteins, 4-α-helical bundle, rop protein, RM6 protein, polypeptide chain directionality, multiangle laser light scattering (MALS), small angle X-ray scattering (SAXS), circular dichroism (CD)

## Abstract

Recurrent protein folding motifs include various types of helical bundles formed by α-helices that supercoil around each other. While specific patterns of amino acid residues (heptad repeats) characterize the highly versatile folding motif of four-α-helical bundles, the significance of the polypeptide chain directionality is not sufficiently understood, although it determines sequence patterns, helical dipoles, and other parameters for the folding and oligomerization processes of bundles. To investigate directionality aspects in sequence-structure relationships, we reversed the amino acid sequences of two well-characterized, highly regular four-α-helical bundle proteins and studied the folding, oligomerization, and structural properties of the retro-proteins, using Circular Dichroism Spectroscopy (CD), Size Exclusion Chromatography combined with Multi-Angle Laser Light Scattering (SEC-MALS), and Small Angle X-ray Scattering (SAXS). The comparison of the parent proteins with their retro-counterparts reveals that while the α-helical character of the parents is affected to varying degrees by sequence reversal, the folding states, oligomerization propensities, structural stabilities, and shapes of the new molecules strongly depend on the characteristics of the heptad repeat patterns. The highest similarities between parent and retro-proteins are associated with the presence of uninterrupted heptad patterns in helical bundles sequences.

## 1. Introduction

As the function of a protein is dependent on its structure, one of the grand challenges in biology since more than half a century has been to understand how proteins fold to create their unique three-dimensional structures. However, despite the employment of experimental protein structure determination techniques of improved accuracy, and the emergence of computational methods to predict protein structures from their amino acids sequences, including refined deep-learning approaches, basic aspects of protein folding and the sequence-structure relationships are still poorly understood.

**Figure 1:**
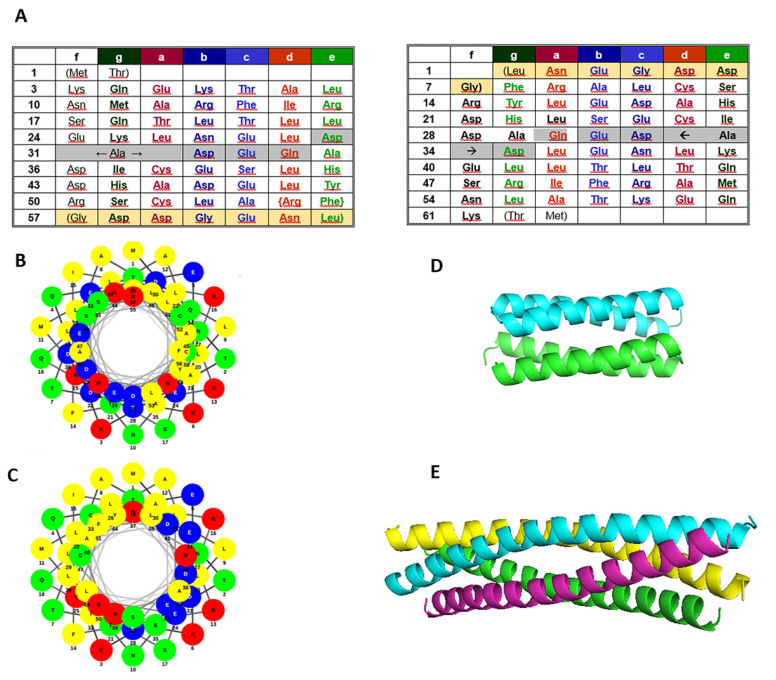
Sequences and available structural data of the proteins examined. (**A**) Rop and RM6 sequences with the assignment of heptad positions (left), rRop and rRM6 sequences with heptad positions assigned in analogy to their parent proteins (right). RM6 and rRM6 lack the residues with grey shading. Light yellow shading indicates disordered residues in Rop and RM6, which do not participate in the coiled-coil structure. Similar shading has been used for their counterparts rRop and rRM6. (**B**,**C**) Helical wheels representation of Rop (**B**) and RM6 (**C**), with polar residues displayed with red (basic), blue (acidic), and green (uncharged) circles, and non-polar residues with yellow. (**D**) Structure of the Rop dimer (PDB id 1ROP). (**E**) Structure of the RM6 tetramer (PDB id 1QX8).

Recurrent motifs of protein structure have been extensively used as model systems for the analysis of protein folding studies and the analysis of motif-specific sequence-structure relationships. In particular, the α-helical coiled-coil motif, one of the most abundant structural protein motifs found in nature, represents a convenient and widely used system, as it combines structural simplicity, remarkable functional versatility, and structural plasticity reflected in a large variety of topologies and folding states (e.g., folded or partially disordered bundles) [1,2,3]. Coiled-coil proteins are bundles of 2–7 α-helices that are coiled together similar to the strands of a rope. They act as principal folding, oligomerization, and intermolecular recognition motifs in proteins and occur either as monomers, or as higher oligomers, e.g., dimers, assembled via oligomerization interactions of α-helical subunits [3]. Computational analysis has established that about 3% of all the amino acids in the known genomes are involved in coiled-coil structures [4]. Coiled-coils are associated with critical functions in almost all the biological systems and processes, e.g., in secretion systems of bacterial pathogens [5], in protein-nucleic acid interactions [1], etc. Basic structural simplicity makes coiled-coils prime candidates for the *de novo* design of proteins or the engineering of building blocks of bio-inspired nano-materials for biotechnology or biomedical applications [6,7]. The amino acid sequences of α-helices in coiled-coil helical bundles are characterized by a pattern of seven-residue quasi-repeats (“heptads”) of the kind (*a*, *b*, *c*, *d*, *e*, *f*, *g*)_n_ [8], where n is the number of repeats and *a*–*g* are the topologically distinct positions of the amino acids in a heptad (Figure 1). The hydrophobic effect is the main driving force for the association and folding of coiled-coils. About 70–75% of the *a*, *d* positions are occupied by apolar residues. The occurrence of hydrophobic residues at the *a*, *d* positions of the heptad repeats is a hallmark feature of coiled-coils. These apolar residues define a hydrophobic stripe which forms the interface between the associating α-helices and the core of the bundle, and pack along it, in a “knobs-into-hole” fashion to minimize their interaction with water molecules [9]. The remaining heptad positions (*b*, *c*, *e*, *f*, *g*) are generally occupied by polar residues, and the stability of coiled-coils is frequently affected by the presence of charged residues at *e* and *g* positions. Detailed position-specific amino acid preferences of four-α-helical bundles have been described by Paliakasis and Kokkinidis [8]. These insights into coiled-coil sequence-to-structure relationships rely to a large extent on protein structure determinations at high resolutions. A typical case is the leucine-zipper motif from the transcriptional activator GCN4, a short peptide of 4.5 contiguous heptads that folds into a stable, parallel, two-stranded coiled-coil of α-helices [10,11], packed as in the “knobs-into-holes” model proposed by Crick [9]. Contacts between the helices include ion pairs and a hydrophobic interface which is formed mostly by leucines and also contains a hydrogen bond. The α-helices cross at −18°, are packed symmetrically, and stabilized by knobs-into-holes interactions between hydrophobic residues of the dimer interface. However, although coiled-coils such as GCN4 have been used traditionally as model systems for protein folding, numerous structural studies of coiled-coils and their mutants have consistently revealed a pronounced structural plasticity [12] that blurs the sequence-to-structure relationships based on the simple concept of heptads periodicity. A need for a better understanding of coiled-coil folding is relevant, due to the importance of this motif in almost all biological processes, and its applications in protein engineering and biotechnology. A particularly informative approach for proteins with significant sequence periodicities, as in the case of helical bundles, is the reversal of their sequences. Due to the structure of the amino acids, a polypeptide chain has directionality, meaning that it has two ends which are chemically distinct from one another, affecting folding differently. Folding studies of proteins with reversed sequences (retro-proteins), can provide insights into the roles of motif-specific structural parameters, e.g., the importance of patterns of specific physicochemical properties in the sequence or the role of helical dipole moments. It should be noted, that generally retro-sequences cannot be aligned with the native sequence [13]. Sequence reversal is also expected to affect secondary structure propensities and α-helix dipole interactions [14], and thus the global stability of the retro protein [15,16]. For coiled-coils in particular, sequence reversal, also affects the native position-specific amino acid preferences of heptad repeats that could potentially prevent the retro protein from properly folding. However, the 35-residue GCN4 leucine zipper fragment which comprises a sequence with a palindromic hydrophobicity pattern and adopts a helical coiled-coil structure, folds into a stable four-helix bundle tetramer [17] in its retro-form. 

Here, we employ sequence reversal to a ubiquitous class of coiled-coils, the 4-α-helical bundles. Structurally, four-helix bundles can occur as an isolated domain or as part of a larger protein. Due to their simplicity relative to other structural motifs, they have served as model systems both for protein folding studies and for the design of novel proteins [18]. Starting from two well characterized bundles, the RNA-binding protein Rop and its 5-residue-deletion variant RM6 [12,19,20], we produced the ‘‘retro-proteins’’ rRop and rRM6 through sequence reversal of Rop and RM6, respectively. Rop and RM6 are paradigms of highly stable and regular 4-α-helical bundles (Figure 1). Rop is a homodimeric, all-antiparallel bundle, with each the monomer forming an α-helical hairpin. The two antiparallel α-helices of the hairpin are connected by a short loop. Thus, compared to GCN4, the monomer of the Rop coiled-coil consisting of a pair of supercoiled, antiparallel helices, is quite different, although the individual helices superimpose rather well between the two coiled-coil structures. Identical helices on the corners of the Rop 4-α-helical bundle are parallel, but these helices are more than 4 Å farther apart than the helices in GCN4. The biological function of Rop is to regulate the plasmid copy number [21,22] through binding to, and stabilizing three transiently formed hairpin pairs between RNA I and its complementary RNA II [23]. The RNA-binding motif forms a narrow, symmetric stripe on one face of the 4-α-helix bundle [24].

At the sequence level (Figure 1), the pattern of heptad repeats of Rop is interrupted only in the loop with a break corresponding either to an insertion of five residues or a deletion of two [20]. RM6 is a Rop variant resulting from the removal of the five residues interrupting the heptad periodicity at the loop region, so that this Rop variant displays an uninterrupted pattern of heptad repeats. The RM6 molecule folds also as a 4-α-helical bundle, which is however completely reorganized relative to Rop, being a homotetramer, with each subunit consisting entirely of a long, uninterrupted α-helix [25]. The helical wheels representation of the sequences of the α-helical segments of Rop and RM6 (Figure 1B,C) show that apolar residues are concentrated overwhelmingly on one side of the helix, as it is also the case with their sequence-reversed counterparts rRop and rRM6 (not shown). The retro-proteins rRop and rRM6 display a pattern of inverted heptads relative to Rop and RM6, respectively. This dictates a re-assignment of positions *a*–*g*, e.g., residues occupying the hydrophobic positions *a* and *d*, are switched to *d* and *a* positions, respectively, in the retro-sequences (Figure 1). For rRop and rRM6, folding studies were performed, and through comparison with Rop and RM6, rules about the folding states of proteins with inverted coiled-coil sequences were deduced.

## 2. Results

### 2.1. Sequence Analysis

Both retro proteins rRop and rRM6 show no significant homologies to other proteins. Similar to their parent proteins Rop and RM6, their hydrophobic-polar residues are organized in a heptads pattern. This pattern is uninterrupted through the entire rRM6 sequence, but it exhibits a discontinuity approximately in the middle of the rRop sequence, through the insertion of the 5-residue peptide QEDAD. This insertion corresponds to the inversion of the peptide DADEQ from the loop region of Rop, which links the two antiparallel α-helices in the α-α-hairpin structure of the Rop monomer. The sequence alignment of both five residue sequences against the PDB database using the BLAST program provides top scores for proteins where the query sequences are usually located in loop regions joining α-helices, and occasionally at the *C*-termini of α-helices or beta sheets which are continued by loops.

### 2.2. Sequence Reversal Affects Differently the Oligomerization Propensities of the Retro-Molecules 

Both rRop and rRM6 were expressed in BL21(DE3) *E. coli* cells using the IPTG induction system. Ni-NTA affinity chromatography purification was performed in the presence of the reducing agent β-mercaptoethanol, as earlier experiments with Rop variants have shown that it promotes the native state over molten globule states or aggregation [12]. 

**Figure 2:**
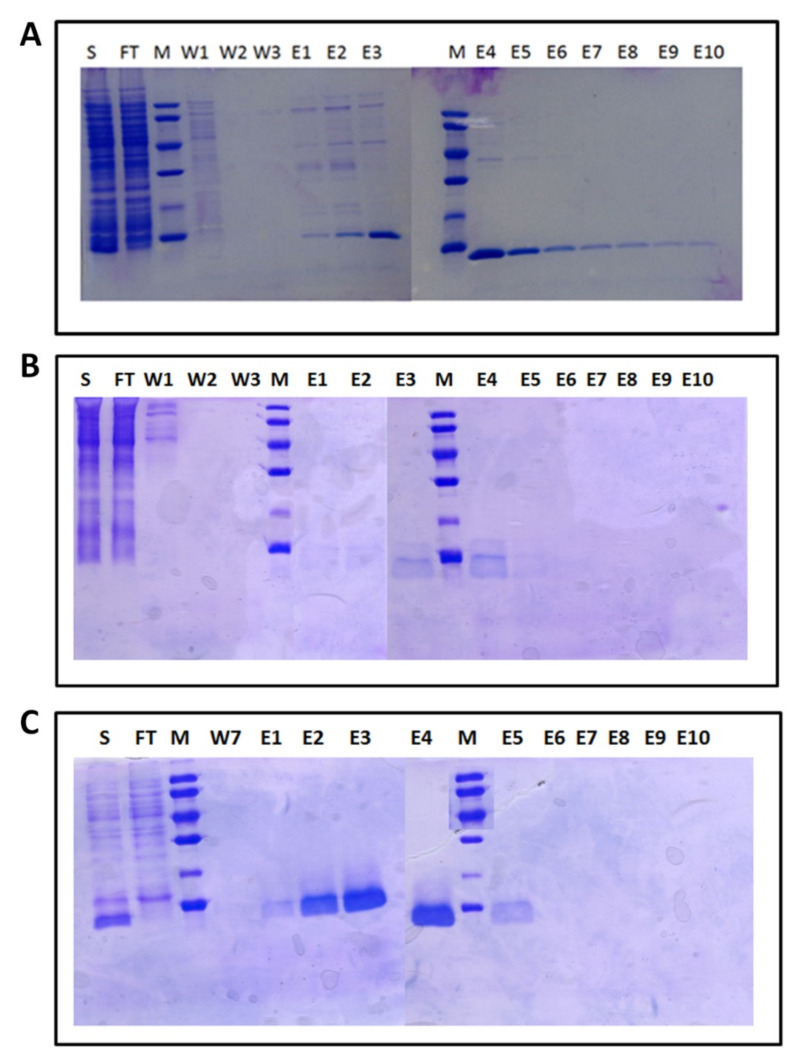
Chromatographic behavior of the retro-proteins monitored by 12.5% SDS gels after the Ni-NTA step of their purification protocols: (**A**) rRM6, (**B**) rRop, non-refolding protocol (Materials and Methods), and (**C**) rRop, refolding protocol. S: Crude extract, the protein sample loaded onto the column (total bacterial cell lysates induced by isopropyl β-d-1-thiogalactopyranoside (IPTG)), FT: Column flow-through (before washing), M: Molecular weight marker (LMW marker, Pharmacia), W: Fractions collected after column washing with increasing imidazole concentrations (10–30 mM), E1–10: Elution fractions obtained with increasing imidazole concentrations (100–300 mM).

**Figure 3:**
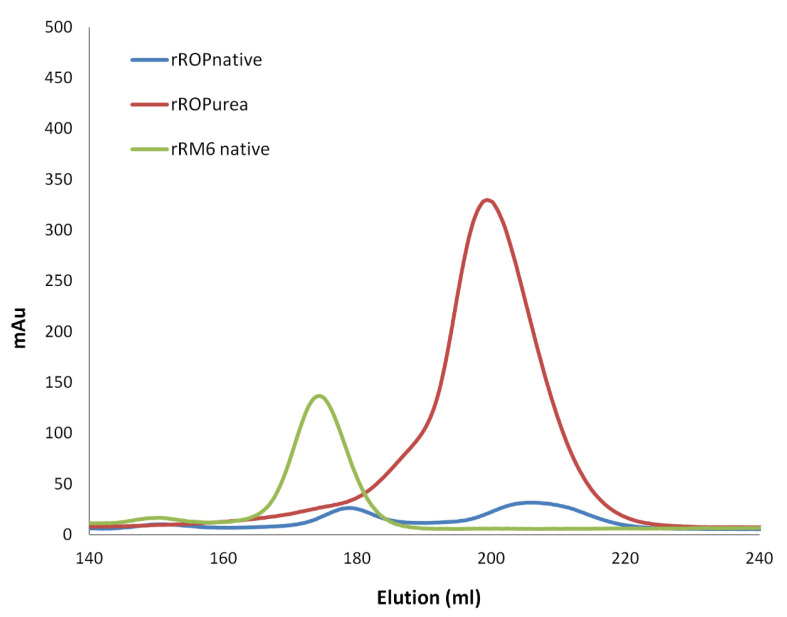
Size Exclusion Chromatography (SEC)elution profiles of the retro proteins. Red: The rRop subjected to urea denaturation in the lysis buffer and subsequent refolding on a Ni-NTA column, prior to SEC purification. Blue: The rRop purified with a Ni-NTA column under non-denaturing conditions, prior to SEC purification. The protein was only detected in the second peak. Green: Elution profile of rRM6 purified under non-denaturing conditions with a Ni-NTA column, prior the SEC purification.

The amount of soluble rRop isolated with the “non-refolding” protocol described in the Materials and Methods section, was insufficient for detailed biophysical and structural characterization (Figure 2). For this protein, a “refolding” protocol using urea was thus developed (Materials and Methods), which yields considerably larger quantities of soluble protein. Subsequent SEC purification of rRop yields an asymmetric peak suggesting polydispersity (Figure 3). The oligomerization state of rRop could not be unambiguously identified as a monomeric or dimeric form. On the other hand, the chromatographic behavior of rRM6 is strikingly similar to that of RM6, suggesting a tetrameric form [25,26] (Figure 2 and Figure 3). 

**Figure 4:**
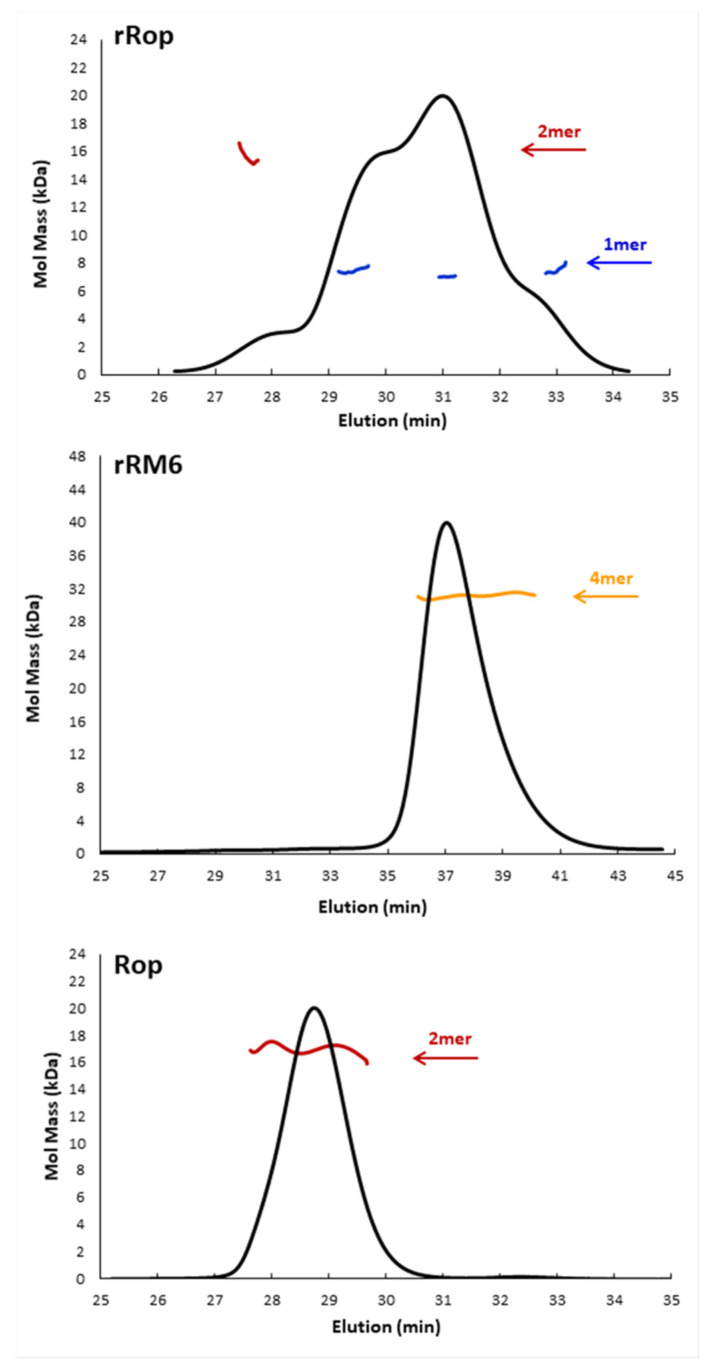
Molecular mass determined by SEC-Multiangle Laser Light Scattering (MALS) for Rop and the retro proteins rRop and rRM6. Shown are profiles of the three proteins. Colored lines correspond to the averaged molecular mass distributions of the eluting species across the peak as determined by MALS. rRop: Blue and red lines correspond to molecular masses of monomers and dimers, respectively. The protein was purified with the non-refolding conditions. rRM6: The yellow line corresponds to a tetramer. Rop: The red line corresponds to the molecular mass of a dimer.

The MALS analysis, confirms the different oligomerization behavior of the two retro-proteins which was observed chromatographically. For rRM6, the presence of a unique tetrameric form suggested by the SEC analysis was confirmed. On the other hand, for rRop purification under non-refolding conditions, the MALS analysis of the main SEC peak, suggests that next to a dominant monomeric peak, a small fraction of rRop molecules exist as dimers (Figure 4). Interestingly, different monomeric populations were detected, suggesting a conformational heterogeneity that could suggest intrinsic disorder or a molten globule-like state. For Rop used as a reference for the MALS analysis, only the dimeric form is detected, in agreement with the X-ray structure of the protein [20].

### 2.3. Sequence Reversal Affects Differently the α-Helical Content, α-Helix Association, and the Stability of the Two Retro-Proteins, While Maintaining Their Overall α-Helical Character

**Figure 5:**
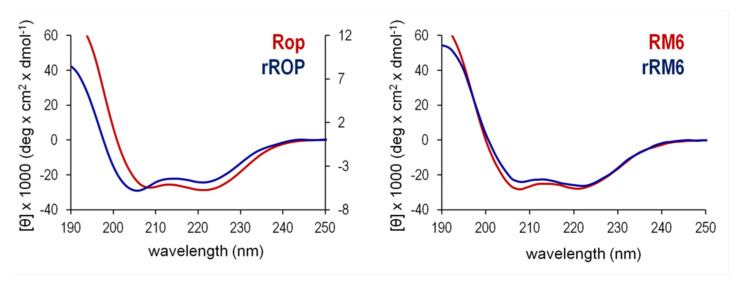
Comparison of the far-UV CD spectra of Rop and RM6 recorded at 20 °C, with those of their retro counterparts. On the left panel, the spectra of Rop and rRop are drawn on a different scale, with the mean residual ellipticity (MRE) values on the left vertical axis corresponding to the CD spectrum of Rop, and the value on the right axis to the CD spectrum of rRop, which was purified using the refolding protocol.

**Figure 6:**
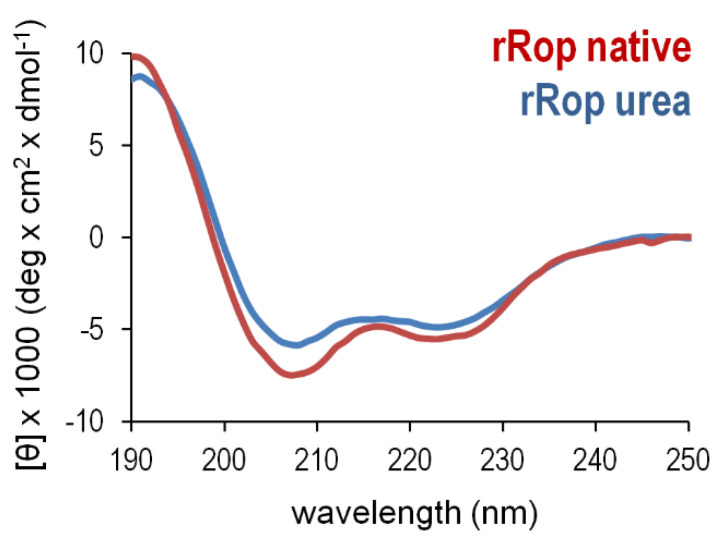
Comparison of rROP samples produced via different purification procedures. CD spectra were collected at 20 °C for one rROP sample purified after urea denaturation and subsequent refolding on a Ni-NTA column (blue), and one sample purified under “non-refolding conditions” (red) as described in the Materials and Methods section.

For both retro proteins, far-UV CD spectra at 20 °C show the characteristic minima at 208 and 222 nm, a distinct signature for α-helical proteins (Figure 5). For rRM6, the CD spectra suggest that despite the absence of sequence homologies, the retro protein exhibits a remarkable similarity at the level of secondary structure to its parent protein RM6, and agrees well with Rop and all other ROP variants studied in the past, which are all-α-helical proteins [12]. The Mean Residual Ellipticity (MRE) values, a measure of secondary structure content [27], also suggest that rRM6 is purely α-helical, comparable to RM6. However, for rRop on the basis of MRE values (Figure 5), a considerably lower α- helical content compared to Rop is suggested. Using the BeStSel method [28] for secondary structure estimation from CD data, we determined a helical content which ranges from 60–67% for rRM6, RM6, and Rop, in agreement with the available structural information (Figure 1). On the other hand, for rRop purified with the non-refolding protocol, the helical content is considerably lower, 5–9% for dilute samples (in the order of 0.3–0.4 mg/mL) and rises to 17% for more concentrated samples (5 mg/mL). In this context, we examined whether the properties of the CD spectra observed for rRop, could depend on the purification protocol used. However, the similarity of the CD spectra from two rRop samples purified under the two different protocols described in Materials and Methods (Figure 6), strongly suggests that their properties are basically independent of the purification strategy.

**Figure 7:**
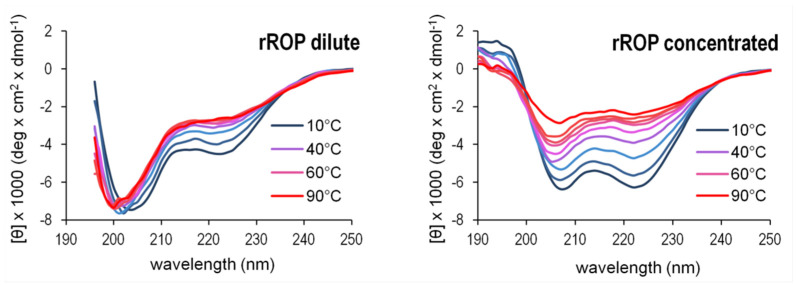
Contribution of protein concentration to α-helix ordering effects in the rRop structure. The concentration effects are monitored by far-UV CD spectra recorded at low (**left**) and high (**right**) protein concentrations for a range of temperatures. The changes of the CD spectra suggest helical ordering effects shifting the spectra from a form associated with non-interacting helices (low rROP concentrations) towards a form associated with a coiled-coil-like structure (high rROP concentrations). For both concentrations, protein samples were purified using the non-refolding protocol.

The θ_222_/θ_208_ ratio of the 222 and 208 nm bands (Figure 5) offers an additional gauge of α-helicity of the two retro proteins: A ratio θ_222_/θ_208_ ≥ 1 characterizes coiled coils, while θ_222_/θ_208_ ≤ 0.86 is expected for isolated helices [29]. The θ_222_/θ_208_ values obtained for the retro proteins are 0.83 (rRop, purified via the refolding protocol), and 1.15 (rRM6). For comparison, θ_222_/θ_208_ is 1.05 for Rop and 1.07 for RM6. These values suggest that rRM6 is a coiled coil comparable to its counterpart RM6 (Figure 5), while rRop, unlike its parent protein Rop, is characterized by a low content of non-interacting α-helices. However, while at low concentrations (0.4 mg/mL) the θ_222_/θ_208_ value for rRop decreases to 0.69 representing a folding state characterized by single-stranded helices, at higher concentrations (5 mg/mL) the ratio is 0.99, which is close to the value for fully folded coiled-coils (Figure 7).

**Figure 8:**
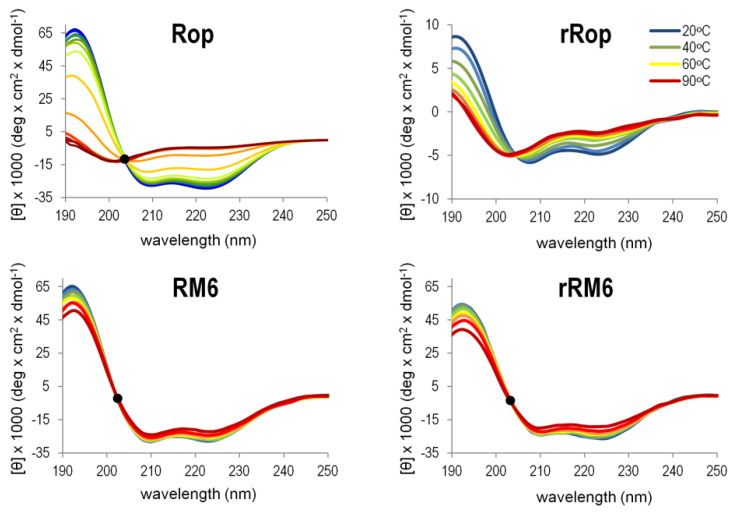
Comparison of the thermal unfolding behavior of Rop, RM6, and their retro counterparts. Thermal unfolding is monitored by far-UV CD scans performed over the temperature range of 20–90 °C. Isodichroic points near 203 nm are depicted by black dots. For rRop, the spectra were collected from protein samples purified with the refolding protocol.

**Figure 9:**
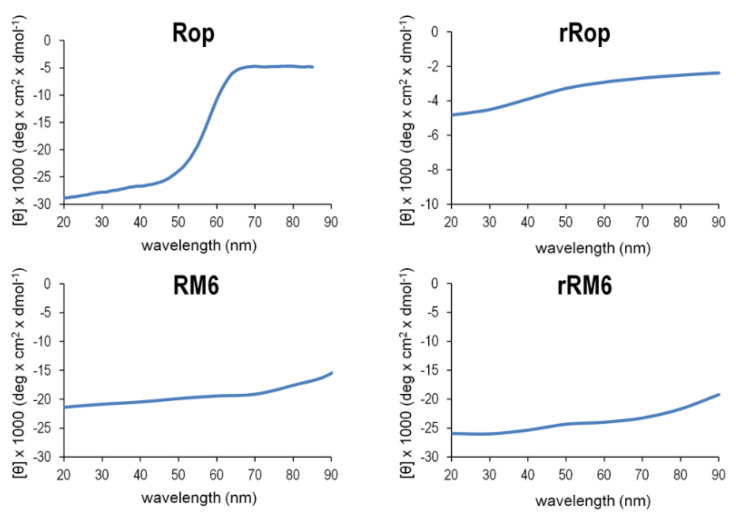
Melting curves of Rop, RM6, and their retro counterparts. Thermal melting curves are obtained from the CD signal at 222 nm in CD spectral scans with increasing temperature (20–90 °C). For rRop, the data were obtained from protein purified with the refolding protocol.

The CD analysis was also used to validate the integrity of the secondary structures of the retro molecules as a function of temperature. The gradual loss of α-helical content with increasingly higher temperatures, was monitored via the change of the two minima of the CD spectrum at 208 and 222 nm, which characterize α-helical proteins (Figure 8). For the thermal unfolding transition, melting curves (Figure 9) were obtained using the temperature dependence of the CD signal at 222 nm. In the temperature range 20–90 °C, rRM6 is a highly stable α-helical protein, with very similar characteristics to those of RM6 which has a T_m_ value of 92 °C. Both proteins retain a very high α-helical content up to the maximum temperature of the experiment and surpass the stability of Rop with exhibits a T_m_ value of 58 °C. The CD spectra of Rop, rRM6, and RM6 are characterized by the presence of an isodichroic point at 203 nm (Figure 8). The existence of an isodichroic point for a given substance indicates a local two-state (α-helix, random coil) population and a two-state folding-unfolding transition. For rRop, the absence of an isodichroic point suggests more complex folding-unfolding behavior. This, along with the fact that rRop has a relatively low content of secondary structure and exhibits little change during thermal denaturation (Figure 9), strongly suggests that this protein is in a disordered, random coil state.

**Figure 10:**
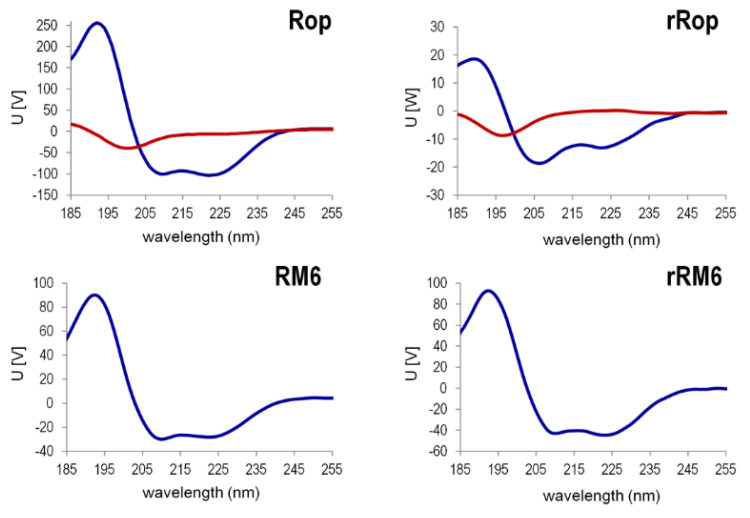
The singular value decomposition (SVD) analysis of the far-UV CD spectra recorded in the temperature range 20–90 °C for Rop, RM6, and their retro counterparts. Only the significant states are depicted (blue-red, in the form of the basis vectors u_i_ weighted by the singular values w_i_. The states shown suffice to model the CD spectra recorded, as all other states are negligible. For rRop, the far-UV CD spectra were from protein samples purified with the refolding protocol.

The Singular Value Decomposition (SVD) analysis [30] (Figure 10) of the far-UV CD spectra (Figure 8) in the temperature range 20–90 °C, reveals that for rRop and Rop, two significant component curves contribute to the CD in the far UV, of which one component curve resembles the CD spectrum of α-helices in a coiled-coil (Rop) or isolated α-helices (rRop). The second component curve is similar to that of polypeptides in a random coil conformation, both for rRop and Rop. For rRM6 and RM6, only one significant component curve is found, corresponding to a coiled-coil α-helix.

### 2.4. SAXS Experiments Reveal the Shapes and Folding States of the Retro Proteins

**Figure 11:**
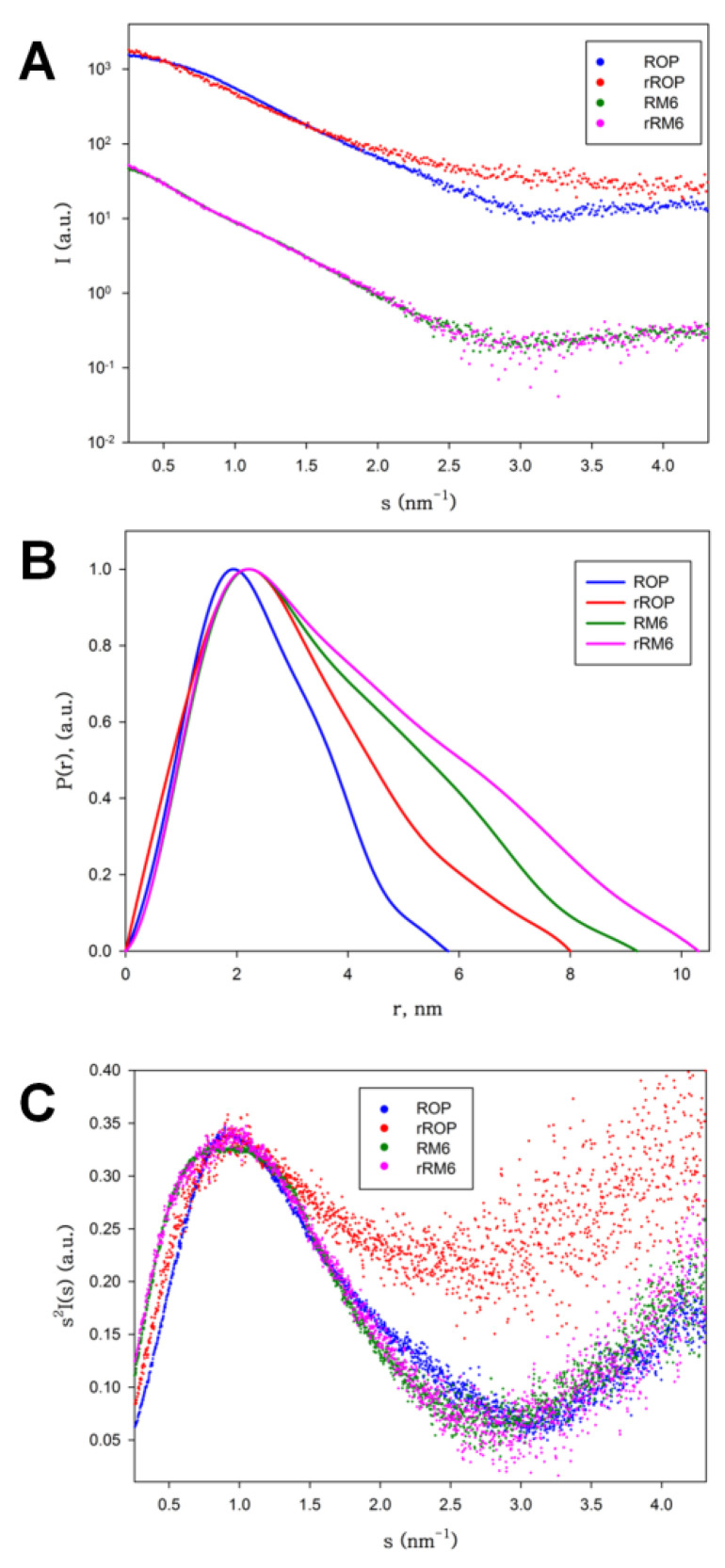
Small Angle X-ray Scattering (SAXS) analysis of Rop, rRop, RM6, and rRM6. (**A**) Scattering patterns, (**B**) distance distribution function P(r) (**C**) Kratky plots. For rRop, the analysis was performed using protein samples purified with the refolding protocol.

Scattering patterns obtained from SAXS experiments (Figure 11) reveal that rRM6 has a very similar shape to RM6, while rRop is significantly different from Rop. The molecular mass estimate of rROP, derived by the Guinier plot I(0) approximation and comparison with a protein standard (BSA), is 7.8 kDa, suggesting the presence of a monomeric population, which is consistent with SEC-MALS results. The estimated molecular mass of rRM6 is 28 kDa, which is consistent with a tetrameric association, also in agreement with the SEC-MALS analysis. RM6 is also a tetramer, an observation also supported by its crystal structure [25]. The radius of gyration (*R*_g_) of rRM6 (3.11 nm ± 0.10) is slightly larger than that of RM6 (2.73 nm ± 0.05), suggesting a more extended shape for rRM6. However, we cannot exclude the possibility of the occurrence of limited aggregation, even at lower concentrations, affecting the *R*_g_ values. On the other hand, rROP has a significantly larger *R*_g_ (2.29 nm ± 0.15) than Rop (1.77 nm ± 0.03), even though Rop is a dimer.

Information about the overall shape of the proteins can be gleaned from the distance distribution functions (P(r)) (Figure 11B). Rop exhibits the expected bell-shaped pattern of a globular protein (maximum size, D_max_ = 5.8 nm), while RM6 (D_max_ = 9 nm) and rRM6 (D_max_ = 10 nm) show skewed distributions expected for rod-like particles compatible with long helical bundles, as has been observed in the RM6 crystal structure [25], supporting once more the significant similarity between these two mutants. The rROP is the most unusual (D_max_ = 8 nm), showing a wider bell-shaped curve indicative of a not very anisometric shape (as it is the case for RM6 and rRM6) but also suggestive of a packing which is less dense for this monomeric protein, compared to the much smaller in size, but dimeric Rop.

The above observations are further reinforced by the Kratky plots, which are indicative of the degree of flexibility of a protein (Figure 11C). The Kratky plot of Rop is consistent with a globular protein, i.e., a sharp maximum, followed by a quick decay of the intensity. RM6 and rRM6 also show this sharp decay but with a broader maximum, consistent with well-folded, rigid, long rod-like particles. In contrast, while rROP also shows a peak, it has a less well-defined shape and a plateau after the peak settles at higher intensities, indicating that the protein has a looser structural packing, and significant flexibility or partial disorder, pointing to a disordered, molten globule-like state. 

## 3. Discussion

### 3.1. The Retro Protein Sequences

Despite sharing with Rop and RM6 the same residues in their heptads and identical global amino acid compositions, the retro proteins rRop and rRM6 are no more similar to their parent protein sequences than to any random sequence. In addition, there are no known sequences with sufficient homology rRop and rRM6 to be considered related. This is consistent with an earlier analysis by Sridhar et al. [31] of the PDB, which could identify identical inverted sequence pairs only for short peptides, ranging in length between 5–12 and 18 amino acid residues. Thus, it cannot be predicted *a priori*, if rRop and rRM6 are foldable, and how stable they are. In fact, there are numerous examples of retro proteins that do not fold at all. On the other hand, the presence of extensive patterns of heptad repeats in the sequences of rRop and rRM6 (although with inverted sequences relative to the heptads of their parent proteins), and our previous work which showed the extreme plasticity of the apolar residues of Rop heptads which can form hydrophobic cores for very different helical bundle topologies [12], suggest that rRop and rRM6 could be foldable. The sequences of the two retro proteins are identical, deviating only by a peptide of five residues, corresponding to the inverted loop region of Rop. This peptide is present in rRop and deleted from the rRM6 sequence. Thus, both retro proteins and their parent proteins represent convenient folding models which can contribute to our global understanding of protein folding and to the folding of coiled-coils in particular.

### 3.2. Chromatographic Behavior and Oligomerization Propensities

In contrast to other retro proteins which are expressed at low levels [13], rRop and rRM6 were expressed at sufficient quantities for further characterization, and were amenable to purification with affinity chromatography under both denaturing, and non-denaturing conditions, and size exclusion chromatography. The rRop and rRM6 differ in their solubilities, with the less soluble rRop requiring a denaturing and refolding procedure for purification in mg quantities. 

SEC, SEC-MALS, and SAXS analyses (Figure 1, Figure 2 and Figure 6) reveal for rRM6 a tetrameric form which is consistent with the results obtained for RM6. On the other hand, for rRop, the prevalent form detected is the monomer, with a small fraction of the protein occurring in the dimeric form, while Rop occurs exclusively as a dimer. Therefore, sequence reversal strongly affects the oligomerization behavior of rRop. A similar discrepancy between the oligimerization states of a retro protein and its parent protein has been observed in the case of the two-stranded helical coiled-coil GCN4 leucine zipper and the retro-GCN4 leucine zipper which forms a tetramer [17]. For both coiled-coil proteins Rop and GCN4 leucine zipper, sequence reversal is equivalent with an *a* → *d* and *d* → *a* transposition in the heptad repeats. For Rop, the four-stranded coiled-coil heptad positions *a* and *d* have different roles in relation to an efficient packing of the α-helices [20]. Thus, it is not surprising, that when residues in positions *a* and *d* are swapped, as is the case upon sequence reversal, the oligomerization state changes, as observed in the rRop and retro-GCN4 leucine zipper. In addition, the reversal of the sequence of the 5-residue peptide at the bend region of Rop, might be expected to affect the oligomerization state of rRop relative to Rop, as the wild-type pattern of hydrophobic and acidic residues of the loop is critical for the assembly and oligomerization of the Rop coiled-coil structure [20]. This pattern is altered upon sequence reversal, thus possibly affecting oligomerization. 

The bend region pentapeptide of Rop and its counterpart in rRop interrupt the regular heptad patterns of the two proteins, breaking the hydrophobicity profiles formed by heptad positions *a* and *d*. In the α-helical hairpin structure of the Rop monomer, the tight constraints of the loop peptide [20] result in two antiparallel hydrophobic stretches, i.e., a geometry of the hydrophobic profile favors the formation of a dimer. As the geometrical constraints of the rRop heptad-pattern-breaking pentapeptide are most likely different, a different geometry of the rRop hydrophobic profile should be expected, resulting in a different oligomerization/aggregation propensity.

The hydrophobicity profile of the RM6 helix results from a long, uninterrupted heptad pattern which is not affected by sequence reversal, thus probably resulting in a nearly identical profile for rRM6, favoring a tetrameric oligomerization mode for RM6 and rRM6.

### 3.3. Secondary Structure, Stability, and Folding States

The far-UV CD spectra of the retro-proteins (Figure 3 and Figure 4) reveal that the secondary structure-forming characteristics of the parent proteins are conserved to varying degrees in the retro proteins. For rRM6, in particular, the secondary structure characteristics revealed by CD are very similar to those shown by the parent polypeptide RM6. Given the extent of the physicochemical changes involved in sequence reversal, it is astonishing that rRM6 folds and assembles into ordered forms exhibiting the same secondary structure characteristics as RM6, a comparable α-helical content, and displays the same thermal unfolding behavior as its parent protein. The extreme structural stability of rRM6 which behaves similar to a hyperthermophilic protein, is comparable to RM6. For both tetrameric proteins, intersubunit interactions, mainly established via their extended, probably geometrically identical hydrophobicity profiles, are expected to play a major role in their extreme stabilities, as they lead to an enlarged buried surface area and reduced flexibility relative to rRop and Rop, which are factors favoring increased thermostability [32]. In this sense, the case of rRM6 which folds into a structure with a qualitatively and quantitatively similar far-UV CD spectrum to that of RM6 is comparable to retro-GroES which has a structure with a β_II_ type of CD spectrum such as GroES [15], or to retro-GCN4-p1, which folds into a helical structure such as GCN4-p1 [17,33].

On the other hand, the far-UV CD spectra of rRop indicate a considerably lower helical content relative to Rop and a θ_222_/θ_208_ ratio, which is characteristic for isolated α-helices, while the Rop spectra are typical for α-helical coiled-coils, in agreement to its known structure. Since rRop was purified via two alternative protocols, involving either refolding or non-refolding conditions, we compared the CD spectra, to verify whether the two different purification protocols elicit the same or different results on the structure of the protein. The rationale is that in principle, different outcomes due to different kinetics are possible between an in vivo folding process (protein synthesized on ribosomes and purified from *E. coli* lysates through non-denaturing affinity chromatography), or an in vitro process (protein synthesis on ribosomes followed by affinity purification combined with a refolding step with the entire polypeptide chain present). Interestingly, nearly identical CD spectra were obtained for rRop both for in vivo and in vitro folding, although for the engineered, sequence-reversed rRop protein, nature did not have the chance to optimize its folding rates and sequence-folding relationships.

It is noteworthy, that as in rRop (Figure 7), protein concentration-dependent effects (molecular crowding) favoring coiled-coil formation, have been also observed in other systems, including synthetic peptides [34] and proteins [35]. The increase in the θ_222_/θ_208_ ratio when the rRop concentration is increased, suggests a transition from a monomeric form (characterized by single-stranded α-helices) to coiled-coil, either intramolecularly within the monomer, or intermolecularly, the latter being consistent with the dimeric form observed by SEC-MALS. Since rRop occurs usually in a monomeric, and probably to a large extent disordered form which is characterized by a relatively low content of non-interacting helices, it may be expected that its buried surface area is reduced, while its structural flexibility/disorder is increased. This is consistent with the differences in the melting curves (Figure 9) between rRop and its parent protein Rop.

### 3.4. Helix Dipoles

It is known that α-helices give rise to dipole moments, oriented along their axes from the *C*- to *N*-termini [36]. These dipole moments result from the alignment of amino and carbonyl groups of α-helical residues, giving rise to a partial positive charge at the *N*- termini and partial negative charge at the *C*-termini of α-helices. The interactions of these dipoles with dipolar or charged groups located at the end of the α-helices have been extensively studied [37,38] and it is accepted that they give rise to specific distributions of amino acid residues at the termini of α-helices [39], with a statistical preference for acidic residues at the *N*-termini of α-helices and basic residues at the *C*-termini. Dipole−dipole interactions within the α-helix increase the preference for α-helix formation and extension of the length of an α-helix by adding additional residues [37]. The inversion of the protein sequences of Rop and RM6, also inverts the direction of α-helix dipoles of rRop and rRM6 relative to their parent proteins. Thus, while Rop and RM6 satisfy the statistical preferences for *N*- and *C*-terminal residues with respect to their helical dipoles, the opposite is true for their retro-proteins. It is not clear whether this has any destabilizing effects, since there is an uncertainty involved in the magnitude of helical dipole interactions, which in comparison with other forces involved in protein folding are expected to be small [40]. The similarities in the physicochemical properties of RM6 and rRM6, despite the opposite direction of their helical dipoles, suggest that electrostatic interactions between helix dipoles are probably not a major driving force for the folding of these proteins, although they may still play a role in the stabilization and extension of the length of α-helices [37].

### 3.5. Sequence Reversal Affects Differently the Folding State of Each Retro Protein

The Guinier plot estimate of the molecular mass, the distance distribution function, and the Kratky plot (Figure 11) obtained from SAXS experiments for rRM6 are compatible with a tetrameric, rigid, well-folded, helical bundle. This result is consistent with the SAXS data obtained for the parent protein RM6 and also with the RM6 crystal structure [25]. A “parent-protein-like” structure is thus a plausible model for rRM6, which is an unusual result for retro-proteins. For comparison, retro-GCN4-p1, which folds into a similar helical structure such as GCN4-p1, deviates from its parent protein at the level of oligomerization. Sequence reversal usually yields proteins that lack thermodynamic stability [13,16,41,42] or proteins that exhibit only residual secondary structure [16], adopt various oligomeric forms [15], or assemble as amyloid fibrils [43]. The simplicity of RM6 that consists at the level of the monomer, of a single α-helix, with an uninterrupted heptad pattern [25], also contributes to the structural and physicochemical similarity to its retro protein rRM6, since in this fold the main chain hydrogen bonds are exclusively formed locally, and elements which are distant in the sequence do not have to interact intramolecularly. In addition, unlike Rop, the RM6 structure is entirely free of loops. These factors correspond to a simple set constraint on the sequence-structure relationships of RM6, and increase the probability that the retro protein adopts a similar folding state as its parent protein. 

On the other hand, rROP significantly deviates in many aspects of its folding bahavior from Rop: At the level of oligomerization, it is predominantly found in a monomeric form, while Rop is always dimeric. The far-UV CD spectra of rRop reflect a folding state characterized by a relatively low, concentration dependent content of secondary structure (α-helical), compared to its parent protein. The concentration dependence of the rRop CD spectra (Figure 7) probably suggest considerable structural plasticity, as rRop switches from a conformation characterized by non-interacting helices, to a coiled-coil-type structure. The absence of an isodichroic point [44] in CD spectra of rRop, points to a non-two-state folding. The SAXS analysis (Figure 11) also supports the presence of a non-native folding state for rRop as it suggests a less dense packing of the molecule in comparison to its parent protein, and a highly flexible, molten-globule state. Generally, molten globules are characterized [45] by the presence of some native-like secondary structure, and a lower compactness of the overall structure of the molecule, properties which agree with the characteristics of the folding state of rRop.

Despite their highly homologous amino acid sequences of their parent proteins, rRop and rRM6 exhibit striking differences in their structural and physicochemical properties. Some characteristics of the Rop and RM6 structures appear to be conserved through the backbone reversal process in the retro-proteins. However, the conservations are significantly more pronounced between RM6 and rRM6, while Rop and rRop exhibit significant differences. The simplicity of the RM6 fold compared to the α-α-hairpin of the Rop structure, appears to create favorable conditions for well folded retro-proteins, which conserve extensively the properties of the parent protein.

The RM6 structure consisting of a single α-helix, the absence of loops, and discontinuities in its heptad repeats pattern RM6, enables specific patterns of amino acid whose physicochemical properties are maintained in both sequences, ensuring that structural features of the original protein are conserved in the retro-protein. In fact, as hydrophobicity is a key driving force in protein folding, the predicted foldability of reversed sequences should depend on the extent to which the parental hydrophobic core is disrupted upon reversal [46], although major rearrangements of hydrophobic cores have been experimentally observed which are compatible with folded coiled-coil structures [12,47]. In this context, cases of de novo protein design have been reported, in which random sequences maintaining a specific pattern of polar and non-polar residues, end-up folding into compact α- helical structures. Thus, the specific pattern of amino acid properties, e.g., heptad repeats, and not solely the sequence can be considered as a major parameter affecting protein folding [48]. This parameter can be efficiently studied through the reversal of protein sequences.

Interestingly, wild-type-like structures and molten-globules have been found for many Rop mutants that maintain the forward sequence direction. Sequence patterns of hydrophobicity, hydrogen bonding, charge, and other amino acid physicochemical properties contribute to their folding mechanisms and structural collapse of the polypeptide chain, even though different helix bundle topologies can be established. The observation that, under conditions in which rRM6 or Rop mutants maintaining forward directionality are found to fold, while rRop adopts a more extended and disordered structure, demonstrates the role for sequence directionality in protein folding, affecting, e.g., the conformations of loop regions. 

## 4. Materials and Methods

### 4.1. Sequence Alignment

Sequence alignments were performed using the NCBI BLAST program BLASTP and the databases GenBank CDS translations (non-redundant), SwissProt, PIR PRF, and PDB.

### 4.2. Synthesis, Expression, and Purification

The rRop and rRM6 genes were synthesized by Genescript. For the attachment of *C*-terminal His_6_ tag, the genes encoding the proteins studied, were cloned into the pET-26b(+) vector (Novagen) and transformed into the *Escherichia coli* strain BL21(DE3). 

For the purification of rRop, a protocol similar to the one described by Kefala et al. [26] was first used, which is referred to as a non-refolding or non-denaturing purification protocol, as no refolding step of denatured protein is involved. As only small quantities of soluble protein could be obtained from the Ni-NTA column, a second purification protocol was developed: 8 M urea was added to the lysis buffer, so as to denature the protein, which was subsequently bound to a Ni-NTA column. To refold rRop, the column was washed, thereby gradually decreasing the urea concentration until it was completely removed. Subsequently, the refolded protein eluted from the column at a concentration of 300 mM imidazole, in quantities which were sufficient for further analysis.

Protein expression and purification of rRM6 followed an earlier, non-refolding, protocol [26]. The production and purification of Rop and RM6 were performed as described earlier [12]: Elution fractions from the affinity chromatography column, containing more than a 90% homogeneous protein, as judged by 12.5% SDS–PAGE gels, were pooled, dialyzed extensively against 25 mM Tris–HCl pH 8.0 100 mM NaCl and 15 mM β-mercaptoethanol in order to remove imidazole, and concentrated using Amicon Ultra-15 filters. The protein was further purified by size exclusion chromatography (SEC) at 20 °C, using an Äkta purifier system (Amersham) and a Sephacryl S-200 high-resolution column (GE Healthcare). The flow rate was 0.5 mL/min, and elution was monitored at 280 nm. Fractions of 2 mL were collected and analyzed using 12.5% SDS-PAGE gels. For all the proteins, final yields were in the order of ∼15 mg pure protein per 10 g of cell paste.

### 4.3. SEC-MALS Analysis

After purification, SEC-MALS—the combination of size-exclusion chromatography with multi-angle light scattering, was used to monitor the oligomerization states of the retro proteins. For rRop, the sample tested was obtained by the non-refolding purification protocol. For all the proteins, the analysis was performed as follows: 100 µL from the samples (wtRop: 3 mg/mL, rRop 5.5 mg/mL, rRM6: 3.5 mg/mL) were loaded onto Superdex 75 (wtRop, rRop) or Superdex 200 (rRM6) columns (GE Healthcare) connected to a high-performance liquid chromatography (HPLC) system (Shimadzu) operating with the LC solution software equipped with a solvent delivery module (Shimadzu; LC-20AD), a UV/VIS photodiode array detector (Shimadzu; SPD-M20A) measuring at 280 nm, a differential refractive index detector (Shimadzu; RID-10A), and a system controller (Shimadzu; CBM-20A) and coupled to online mass detection by an advanced 8 angles MALS detector (Wyatt; Dawn 8+) with an integrated Wyatt QELS Dynamic Light Scattering (DLS) module. Data were analyzed with the Astra software (ASTRA 6.1.2.84). 

### 4.4. Circular Dichroism Measurements

Far-UV CD spectra (180–250 nm) for RM6, rRM6, and rROP were collected using synchrotron radiation on the DISCO beamline at the SOLEIL synchrotron in France. CD spectra for wtRop (190–250 nm) were collected using a J-810 CD spectropolarimeter (Jasco Inc., Easton, MD, USA). Thermal denaturation was analyzed in the range of 20–90 °C in steps of 10 °C and monitoring the change of the typical α-helical minima at 208 and 222 nm. Melting curves were obtained from the change of the CD signal at 222 nm for temperatures 20–90 °C. The protein concentration was 4 mg/mL for wtRop, 5 mg/mL for RM6, 12.5 mg/mL for rRop, and 7 mg/mL for rRM6 in 25 mM Tris pH = 8, 100 mM NaCl, and 15 mM β-mercaptoethanol. CaF_2_ cuvettes of 12 µm path length were used in all cases of measurements at DISCO beamline, whereas a 1 mm quartz cuvette was used for the JASCO system. The beamline software was used for buffer subtraction and unit conversions to mean residual ellipticities (MRE). 

CD spectra for rRop were also obtained using a J-810 CD spectropolarimeter (Jasco Inc.) with quartz cuvettes of 1-mm path length and a protein concentration of 0.4 mg/mL or at 5 mg/mL using a 0.1-mm demountable quartz cuvette. Far-UV spectra (190–250 nm) were recorded at a 50-nm/min scanning speed, 2-min response time, and three accumulations. Thermal denaturation was monitored by the change of the CD signal at 222 nm for 10–90 °C with the temperature increasing 80 °C/h and a waiting time of 2 s for stabilization. The Spectra Manager program (Jasco Corp.) was used for buffer subtraction and unit conversions to MRE. 

The singular value decomposition (SVD) [30] of the far-UV CD spectra with the program SVD1 [49], determined the significant independent states of the unfolding transition. As reported for other proteins [5], the unfolding process can be modeled with the significant species determined by SVD on the basis of the characteristics of the SVD basis vectors (U), and the temperature dependence of their associated coefficients (V). 

### 4.5. SAXS Measurements

SAXS data were collected at the EMBL Hamburg P12 undulator beamline of the Petra III storage ring in DESY (Hamburg, Germany) using a Pilatus 2M (DECTRIS) photon counting pixel detector [50]. The measurements were performed at 10 °C at different concentrations from 1–12 mg/mL using the automated sample changer. The sample-to-detector distance was 3.1 m, covering a range of momentum transfer 0.02 < *s* < 4.8 nm^−1^ (*s* = 4*π* sin*θ*/*λ*, where 2*θ* is the scattering angle, and λ = 1.24 Å is the X-ray wavelength). Primary data reduction, radial averaging, averaging and subtraction were performed on-site with the beamline software (SASFLOW, v. 3.0, Hamburg, Germany). A subsequent analysis was performed with the ATSAS program suite [51]. PRIMUS [52] was used for the calculation of the radius of gyration *R*_g_ and the forward scattering intensity *I*(0) from the slope of Guinier plot (ln*I*(*s*) vs. *s*^2^) [53]. The molecular mass (MM) of the solute was estimated from the SAXS data from the *I*(0). GNOM [54] was used to calculate the pair distance distribution function *p*(*r*) and to estimate the maximum particle dimension (*D*_max_). The flexibility and anisometry of the proteins was assessed with Kratky (*s*^2^*I*(*s*)/*I*(0) vs. *s*) plots [55].

## 5. Conclusions

Although nearly 50 years ago Christian Anfinsen was awarded a Nobel Prize for showing that the shape of proteins is determined by their sequence of amino acids, protein folding is still poorly understood. Today, there is a wide range of theoretical and experimental approaches to this problem, all with a different focus, from a general understanding of the protein fold, to more detailed predictions of side-chain configurations. Amino acid sequence reversal is one potentially powerful and informative approach since, as the retro protein sequences are distant from other naturally occurring proteins, they offer insights into sequence-structure relationships not yet sampled by nature. On the other hand, despite the absence of any sequence homology between parental and retro sequences, some features of the original protein, such as its amino acid composition and general patterns of physicochemical properties [13,46,56] are maintained in the reversed sequence. Therefore, retro-proteins were suggested to be more “foldable” than random sequences and the folding conservation between the parental and retro-protein has been frequently suggested by modeling or theoretical studies [57,58]. However, experimental studies have contradicted these predictions, necessitating broader studies of retro proteins.

The Rop structure has provided for several years a convenient model system for folding studies focused on one recurrent motif of protein structure, the coiled-coil architecture. Yet, despite the availability of atomic resolution models for wild-type Rop and several of its mutants, no satisfactory understanding of how this model system folds has been yet developed.

The retro proteins studied in this work represent simple folding models which, being foldable, contribute considerably to our global understanding of coiled-coils folding, by providing access to parts of the sequence space which are not used by known natural proteins for coiled-coil folding. The structural plasticity and the foldability of these proteins adds them along with Rop and RM6 to the list of molecules which are potentially suitable for the engineering of novel, bio-inspired materials.

Our work is the first protein folding study that approaches systematically highly homologous α-helical coiled-coils through reversal of their amino acid sequences. Further structural and computational analyses of these retro proteins will provide even more detailed insights into the effects of sequence directionality on protein folding and oligomerization.

## Abbreviations

MALS	Multiangle Laser Light Scattering
MRE	Mean Residue Ellipticity
SAXS	Small Angle X-ray Scattering
SEC	Size Exclusion Chromatography
CD	Circular Dichroism
PDB	Protein Data Bank
